# The Historical Demography and Genetic Variation of the Endangered *Cycas multipinnata* (Cycadaceae) in the Red River Region, Examined by Chloroplast DNA Sequences and Microsatellite Markers

**DOI:** 10.1371/journal.pone.0117719

**Published:** 2015-02-17

**Authors:** Yi-Qing Gong, Qing-Qing Zhan, Khang Sinh Nguyen, Hiep Tien Nguyen, Yue-Hua Wang, Xun Gong

**Affiliations:** 1 Plant Science Institute, School of Life Sciences, Yunnan University, Kunming, Yunnan, China; 2 Key Laboratory for Plant Diversity and Biogeography of East Asia, Kunming Institute of Botany, Chinese Academy of Sciences, Kunming, Yunnan, China; 3 University of Chinese Academy of Sciences, Beijing, China; 4 Institute of Ecology and Biological Resources, Vietnam Academy of Science and Technology, Cau Giay District, Ha Noi, Vietnam; 5 Center for Plant Conservation, Cau Giay District, Ha Noi, Vietnam; Università Politecnica delle Marche, ITALY

## Abstract

*Cycas multipinnata* C.J. Chen & S.Y. Yang is a cycad endemic to the Red River drainage region that occurs under evergreen forest on steep limestone slopes in Southwest China and northern Vietnam. It is listed as endangered due to habitat loss and over-collecting for the ornamental plant trade, and only several populations remain. In this study, we assess the genetic variation, population structure, and phylogeography of *C. multipinnata* populations to help develop strategies for the conservation of the species. 60 individuals from six populations were used for chloroplast DNA (cpDNA) sequencing and 100 individuals from five populations were genotyped using 17 nuclear microsatellites. High genetic differentiation among populations was detected, suggesting that pollen or seed dispersal was restricted within populations. Two main genetic clusters were observed in both the cpDNA and microsatellite loci, corresponding to Yunnan China and northern Vietnam. These clusters indicated low levels of gene flow between the regions since their divergence in the late Pleistocene, which was inferred from both Bayesian and coalescent analysis. In addition, the result of a Bayesian skyline plot based on cpDNA portrayed a long history of constant population size followed by a decline in the last 50,000 years of *C. multipinnata* that was perhaps affected by the Quaternary glaciations, a finding that was also supported by the Garza-Williamson index calculated from the microsatellite data. The genetic consequences produced by climatic oscillations and anthropogenic disturbances are considered key pressures on *C. multipinnata*. To establish a conservation management plan, each population of *C. multipinnata* should be recognized as a Management Unit (MU). *In situ* and *ex situ* actions, such as controlling overexploitation and creating a germplasm bank with high genetic diversity, should be urgently implemented to preserve this species.

## Introduction

The Cycad is considered an old lineage because of its ancient morphological characters and fossil records which could date to the Early Permian [1] or possibly even the late Carboniferous period (approximately 300 million years ago) [2]. Cycads became a dominant plant group during the Mesozoic, as shown by numerous fossils of megasporophylls and ovalate strobili, as well as vegetative shoots, leaves and trunks [3–9]. However, one recent fossil-calibrated molecular phylogenetic study based on multiple DNA sequence data proposed that extant Cycads originated no more than 12 million years ago; the Cycads underwent a recent synchronous global rediversification beginning in the late Miocene, followed by a slowdown towards the recent [10].

*Cycas*, as the basal lineage of the living cycads supported by both phylogenetic studies and the recent obtained genome size of nuclear DNA [11–14], is the sole genus of Cycadaceae, and one distinguishing character of *Cycas* from other Cycads in morphology is its leaflets with an obvious midrib, lacking lateral veins. The leaf of *Cycas* is pinnate or rarely bipinnate, and *Cycas multipinnata* C.J. Chen & S.Y. Yang is one of the four species characterized by the latter [15]. The somatic chromosome number of *C*. *multipinnata* is 2n = 2x = 22 [16], and the karyotype is classified as 3B according to Stebbin’s category.

*Cycas multipinnata* distributes in the Red River drainage zone under an evergreen forest canopy on the sharp limestone slopes of southwest China (Yunnan Province) and northern Vietnam (Yen Bai and Tuyen Quang Province) [17,18]. The Red River (also called the Yuanjiang River in China and Song Hong in Vietnam) flows for 692 kilometers in China, southeastward across Hekou County into northern Vietnam, before emptying into the sea. The area, through which the Red River flows, in geology terms, is known as the Red River fault zone. The collision of the India Plate with Asia caused the Indochina Plate to move approximately 700 km southeast relative to the South China Block along the Red River fault line [19,20]. At least 24 *Cycas* species occur in this small region, which ranges from the coast of Vietnam well into the interior of China along the Red River. *Cycas multipinnata* is one of the 17 *Cycas* species endemic to this area [21]. However, the wild individuals of *C*. *multipinnata* in both China and Vietnam declined dramatically in recent decades due to the anthropogenic disturbances, particularly habitat loss and over-exploitation for the ornamental plant trade. *Cycas multipinnata* was assessed as Endangered (EN) by the IUCN (the International Union for the Conservation of Nature) Categories and Criteria system [21,22].

Chloroplast DNA of *Cycas* is maternally inherited [23], so it is dispersed by seeds; its nuclear microsatellite is biparental inheritance, which is dispersed via both seeds and pollen. In this study, three intergenic spacers of chloroplast DNA and 17 microsatellite loci were employed to assess the genetic diversity, the structure and the population dynamics of *C*. *multipinnata*. Further, we discuss the causes of the genetic consequences, such as an environment with climatic oscillations or human impacts. These results show the urgency and significant implications of the management and recovery of this endangered species.

## Materials and Methods

### Ethics Statement

*Cycas multipinnata* is the First Grade conservation plant in China [24]. It was assessed as Endangered (EN) by the IUCN (the International Union for the Conservation of Nature) [22]. We got the permission of the Wildlife Protection and Administration Office under the Forestry Department of Yunnan, China and the permission of owners for some private cultivated plants. There is cooperation agreement between the Center for Plant Conservation (CPC) of Vietnam Union of Science and Technology Association and the Kunming Institute of Botany (KIB) of the Chinese Academy of Sciences on the joint exploration to the flora of globally threatened plant. We also got the permission of local forestry department in Vietnam when collecting the *Cycas* samples in the wild. The sampling process was under the guidance of local rangers. Our sampling will not affect the regular growth of *C*. *multipinnata*, and it was solely used for scientific research.

### Population sampling and DNA extraction

**Fig 1:**
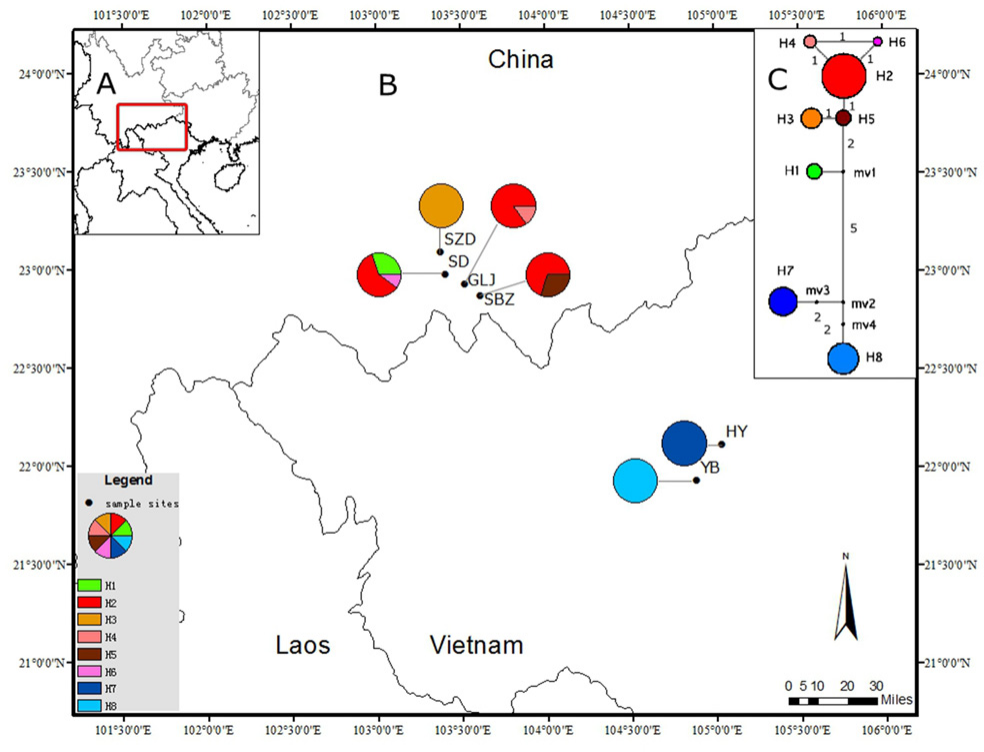
Geographic distribution and evolutionary relationships of the eight cpDNA haplotypes in *Cycas multipinnata*. A: General position of map B; B: Pie charts show the proportions of haplotypes within each population. C: The network of the eight cpDNA haplotypes. The numbers on the branches represent the number of mutations between two connected haplotypes and black dots (mv) represent missing haplotypes (not sampled or extinct).

A total of 105 individual samples were collected from six populations of *C*. *multipinnata* (four populations, including a cultivated SD population in a village because the nearby wild habitat was totally destroyed, were sampled in Yunnan Province, China and two populations were sampled in northern Vietnam). All six populations were located north of the Red River. Of the 105 samples, 60 individuals from the six populations were used for chloroplast DNA sequencing. The population known as SZD was eliminated from SSR analysis because there were only five individuals in the population. A total of 100 individuals from five populations were used for the microsatellite study. Information on each sampling location and the number of individuals from each population that were used in DNA sequences and SSR analysis is presented in Table 1 and Fig. 1, respectively.

**Table 1:** Information on sampling sites of *Cycas multipinnata*.

Population(sampling site)	Code	Latitude (N)	Longitude (E)	Altitude (m)	N(for SSR)	Hd	π
Shi dong, Yunnan, China	SD	22°59′	103°24′	254	10(20)	0.6000	0.00072
Shibanzhai, Yunnan, China	SBZ	22°53′	103°37′	580	10(20)	0.4667	0.00024
Ganlongjing, Yunnan, China	GLJ	22 °56′	103°31′	600	13(20)	0.2821	0.00014
Shazudi, Yunnan, China	SZD	23°06′	103°23′	1000	5(0)	0	0
Ham Yen, Tuyen Quang, Vietnam	HY	22°07′	105°02′	50	12(23)	0	0
Yen Binh, Yen Bai, Vietnam	YB	21°56′	104°53′	225	10(17)	0	0

N: the number of individuals for cpDNA sequencing, and the number for SSR genotyping

Healthy leaves were dried and preserved in silica gel immediately after being collected. Genomic DNA was extracted from the leaves, which was ground into a powder after being frozen with liquid nitrogen, following the cetyltrimethyl ammonium bromide (CTAB) method [25]. The total DNA was dissolved in TE (Tris-EDTA) buffer and used as the template in the polymerase chain reaction (PCR).

### Chloroplast DNA analysis

Three cpDNA regions *atp*B-*rbc*L, *psb*A-*trn*H and *psb*B-*psb*H were selected. The PCR amplifying conditions and primers of *atp*B-*rbc*L and *psb*A-*trn*H were identical to those described by Zhan [26]. The primers and cycles of *psb*B-*psb*H fragment were as follows: *psb*B (F) 5′-TCCAAAAACTGGGAGATCCAAC-3′, *psb*H (R) 5′-TCAATGGTCTGTGTAGCCAT-3′ [27]; one cycle of four min at 80°C, 30 cycles of 50 s at 94°C, 50 s at 52°C, and 50 s at 72°C, with a final extension step for eight min at 72°C.

Purified PCR productions were sequenced in both directions using the same primers for the amplification reactions, using an ABI 3730 automated sequencer at Shanghai Majorbio Bio-pharm Technology Co., Ltd.

### Microsatellite genotyping

Microsatellite primers of *Cycas* have been developed by several research groups. 17 variable microsatellite loci were selected from the published 65 microsatellite loci [28–34] that developed in other species of *Cycas* to investigate the genetic diversity of *C*. *multipinnata* (Table A in S1 File). The PCR amplification was performed in a 25 μL reaction, with the forward primers labeled with fluorescent dye (FAM, TEMRA or HEX) and visualized on an ABI 3730xl Capillary DNA Analyzer by Sangon Biotech (Shanghai) Co., Ltd. Fragment sizes were assessed using GeneMapper version 4.0.

### Data analysis of cpDNA

After being edited and jointed in the SeqMan of software package (DNA Star), sequences were aligned using Clustal X version 1.81 [35], and fine adjustments were performed manually using the BioEdit version 7.0.1 [36] software. The incongruence length difference test was conducted using the partition homogeneity test in PAUP version 4.0b10 [37] to examine the congruence between datasets. Insertions and/or deletions were coded as binary characters. All character states were specified as unordered and equally weighted.

DnaSP version 5.10 [38] was used to calculate the number of haplotypes, variable sites and nucleotide diversity per site (π). A map of the geographic distribution of haplotypes was drawn via ArcGIS 10.2 [39]. Permut 1.0 (http://www.pierroton.inra.fr/genetics/labo/Software/Permut) was used to calculate the within-population diversity (H_S_), the total diversity (H_T_) and two measures of population differentiation, G_ST_ and N_ST_ [40]. Arlequin version 3.11 [41] was used for the molecular variance (AMOVA) analysis [42] to estimate genetic variations within and among populations.

We calculated the genetic distance (GD) among the six populations following the formula GD = F_ST_/ (1-F_ST_) [43] using Arlequin version 3.11 [41]. Geography distance (GGD) was calculated via GenAlEx version 6.4.1 [44]. A Mantel test was designed to test the relationship of the elements of two matrices in GenAlEx version 6.4.1 [44].

The genealogical haplotype network was constructed using Network version 4.6.1.2 (http://www.fluxus-engineering.com/sharenet.htm) following the media-joining calculation [45]. Phylogenetic relationships among the cpDNA haplotypes were reconstructed by Maximum Parsimony (MP), Neighbor Joining (NJ) and Maximum Likelihood (ML) analyses using PAUP version 4.0b10 [37] and Bayesian methods implemented in MrBayes version 3.1.2 [46], with *C*. *panzhihuaensis* serving as the outgroup. BEAST version 1.7 [47] was used to estimate the ages of the most recent common ancestor (TMRCA). The analysis was run for 10^7^ iterations with a burn-in of 10^6^ under the HKY (Hasegawa-Kishino-Yano) nucleotide substitution model, which was determined to be the most suitable model by Modeltest in Mega 6.06 [48] and a strict molecular clock. A well-documented evolutionary rate for cpDNA, 1.01×10^-9^ substitution per site per year [49,50] for synonymous sites, was used to estimate the coalescent time between lineages across haplotypes.

A Bayesian skyline plot was constructed by BEAST version 1.7 [47] and TRACER version 1.5 [51] to infer the past population dynamics. To infer the possible demographic expansion of *C*. *multipinnata*, mismatch distribution analysis based on the sudden population expansion model using the observed number of differences between pairs of haplotypes were conducted with DnaSP version 5.10 [38]. The sum of squared deviations (SSD) between the observed and expected mismatch distributions, the raggedness index (HRag) and their significance [52] were calculated in Arlequin version 3.11 [41]. We also conducted neutrality tests, with Tajima’s *D* [53], Fu and Li’s F*& D* [54], as well as Fu’s *F*_*S*_ [55], using Arlequin version 3.11 [41], to detect departures from the population equilibrium.

### Microsatellite fingerprinting analysis

The dataset was edited and transformed to other formats in GenAlEx version 6.4.1 [44]. We tested for evidence of selection on each locus using LOSITAN [56], which can detect excessively high or low F_ST_ compared with neutral expectations. Deviations from Hardy-Weinberg equilibrium (HWE) could indicate the presence of population structure or inbreeding [57]. HWE was tested for each locus and each population with default parameters using Genepop version 4.1.4 [58]. Linkage disequilibrium was investigated at the 5% statistical significance level among loci pairs with 1000 permutations using Arlequin version 3.11 [41].

The indices of genetic diversity within populations, such as the number of alleles (N_T_), the number of private alleles (A_P_), the mean number of alleles (N_A_), the effective number of alleles (N_E_), the observed heterozygosity (H_O_), the expected heterozygosity (H_E_), the information index (I), the fixation index (F) and the percentage of polymorphic loci (PPB) were calculated using GenAlEx version 6.4.1 [44]. Differentiation between pairs of populations was computed using F_ST_ and tested with GenAlEx version 6.4.1. Allelic richness (A_R_) was estimated with FSTAT, version 2.9.3) was estimated with FSTAT, version 2.9.3 [59].

As in the analysis of cpDNA, genetic distance (GD) and geography distance (GGD) among the five populations was calculated using the Arlequin version 3.11 [41] and GenAlEx version 6.4.1 [44], respectively. A Mantel test was designed to test whether this species was isolated by distance (IBD) using GenAlEx version 6.4.1 [44]. Gene flow between pairs of populations was estimated based on Wright’s principles [60], *N*m = (1-F_ST_)/4 F_ST_.

An individual-based principal coordinate analysis (PCO) was conducted in the MVSP version 3.12 software [61] using the genetic distances among SSR genotypes. The PCO could visualize genetic relationships among these 100 individuals from the five populations. We also conducted a Bayesian analysis of population structure on the SSR data using STRUCTURE version 2.3 [62]. The admixture model was used and the posterior probability of the grouping number (*K* = 1~8) was estimated by the Markov chain Monte Carlo (MCMC) method with 10 separate runs to evaluate the consistency of the results. Each run was estimated as 100,000 steps, with a 100,000-step burn-in. The best fit number of grouping [63] was evaluated using Δ*K* in the STRUCTURE HARVESTER v. 0.6.93 tool [64]. Finally, we identified geographical locations where major genetic barriers among populations might occur with a barrier boundary analysis using BARRIER version 2.2 [65], based on genetic distance matrices.

A heterozygosity excess test at the population level from BOTTLENECK 1.2.02 was used to detect the recent population bottleneck [66]. The computation was performed under the two phased model (TPM) [67]. Two methods, the Sign test and the Wilcoxon test, which are powerful and robust statistics when using less than 20 polymorphic loci, were executed in the model. Second, we used Bottleneck to examine the distribution of the alleles’ frequencies for a so-called mode-shift that discriminates recently bottlenecked from stable populations. The method implemented in the bottleneck has low power [68] unless the decline is greater than 90%, so we computed the Garza-Williamson index, a statistic that can detect population bottlenecks using Arlequin version 3.11. The Garza—Williamson index [69] is the mean ratio of the number of alleles at a given locus to the range in allele size, i.e., *M* = (*k*/*r*), where *k* is the number of alleles and *r* is the allelic range (i.e., the difference in repeat units between the shortest and the longest alleles at a locus). This measure is based on the assumption that in a bottleneck event, the number of alleles decreases faster than the allelic range because the latter is only reduced if the shortest and/or longest allele is lost, whereas the loss of any allele reduces the former. The Garza and Williamson found critical values for *M* < 0.68, which indicated a bottleneck, and *M* > 0.80, which indicated no reduction of effective population size.

Effective population sizes (Ne) are among the most important parameters in wildlife management and conservation because they can inform management and help predict the extinction risk of populations. We estimated effective population sizes (Ne) using the linkage disequilibrium (LD) method in LDNe [70] at three levels of lowest allele frequency (0.01, 0.02, 0.05) for a 95% confidence interval.

## Results

### Sequence variation and genetic diversity of cpDNA

The three cpDNA fragments, *atp*B-*rbc*L (delete the poly T), *psb*A-*trn*H and *psb*B-*psb*H, are 719, 597 and 644 bp in length, respectively (GenBank accession numbers: KP335666—KP335683). The partition-homogeneity test indicated that data sets of these cpDNA fragments are significantly congruent (P = 1). The 1960 bp combined cpDNA data set had 14 parsimony-informative polymorphic sites and 8 haplotypes (H1–H8) (Table B in S1 File). The haplotype frequencies in each population and geographical distribution are presented in Fig. 1. H2 is the most abundant haplotype. The two populations in Vietnam have only one unique haplotype, respectively. The haplotype diversity (H_d_) is 0.7718, and the nucleotide diversity per site (π) is 0.00149. The total diversity (H_T_) is 0.896, and the within-population diversity (H_S_) is 0.225.

Significant population differentiation was observed, with a G_ST_ = 0.749 and an N_ST_ = 0.922, and the permutation test showed that G_ST_ and N_ST_ were not significantly different from each other (N_ST_ > G_ST_, P > 0.05). The results of the AMOVA analysis indicated that 92.3% variation occurs among the populations (Table 2), with F_ST_ = 0.92304.

**Table 2:** Results of Analysis of Molecular Variance (AMOVA, cpDNA/nSSR) of *C*. *multipinnata*.

Source of variation	d.f.	Sum of squares	Variance components	Percent of variation (%)
Among populations	5/4	144.774/228.593	2.90808/1.35128 Va	92.304/29.57
Within population	54/195	13.092/627.642	0.24254/3.21868 Vb	7.696/70.43

d.f.: degree of freedom

**Fig 2:**
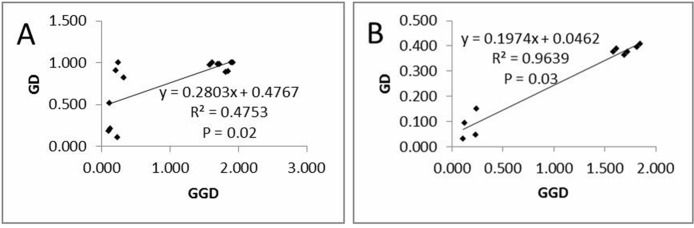
Scatterplots showing the relationship between genetic distance (GD) and geographic distance (GGD) based on cpDNA (A) and SSR data (B).

The Mantel test (Fig. 2A) showed that there was a significant positive correlation (P < 0.05) between the genetic distance (GD) and the geographic distance (GGD), suggesting that *C*. *multipinnata* was isolated by distance.

### Phylogenetic inferences based on the combined cpDNA sequence data

**Fig 3:**
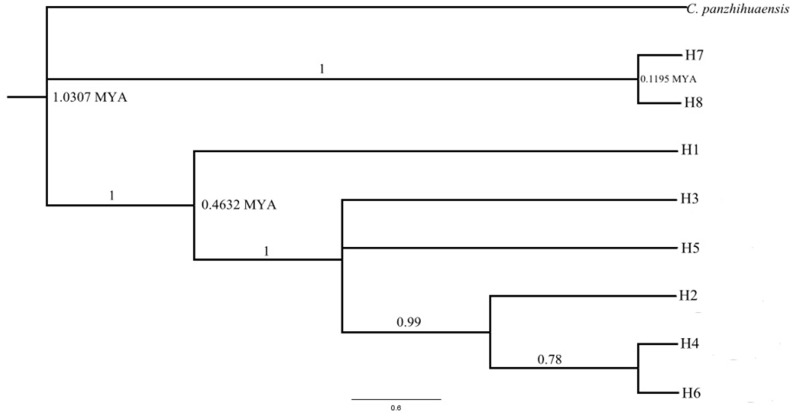
Phylogenetic relationships of the cpDNA haplotypes based on the Bayesian analysis.

The genealogical haplotype network is shown in Fig. 1(C). In this figure, we can observe that each of the two populations in Vietnam has a unique haplotype. Additionally, there were at least eight mutations between the haplotypes (H7 or H8) in Vietnam and haplotypes (H1–H6) in China. The topology of the Bayesian tree of the eight haplotypes detected from the combined cpDNA of *C*. *multipinnata*, with *C*. *panzhihuaensis* as outgroup, is shown in Fig. 3, and the major clades were the same as the topology of MP and ML trees. Two clades were identified in the tree and the network. Clade I contained two haplotypes (H7, H8) from the northern Vietnam, and clade II included the remaining haplotypes that occurred in Yunnan provinces, China. The date of the most recent common ancestor (TMRCA) of the China clade and Vietnam clade was approximately 1.0307 million years ago (MYA).

### Demographic analysis of the cpDNA

**Fig 4:**
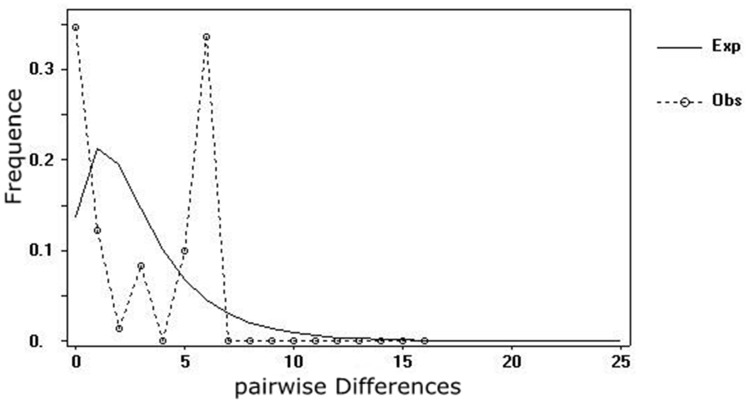
Mismatch distributions of cpDNA haplotypes based on pairwise sequence difference plotted against the frequency of occurrence.

**Fig 5:**
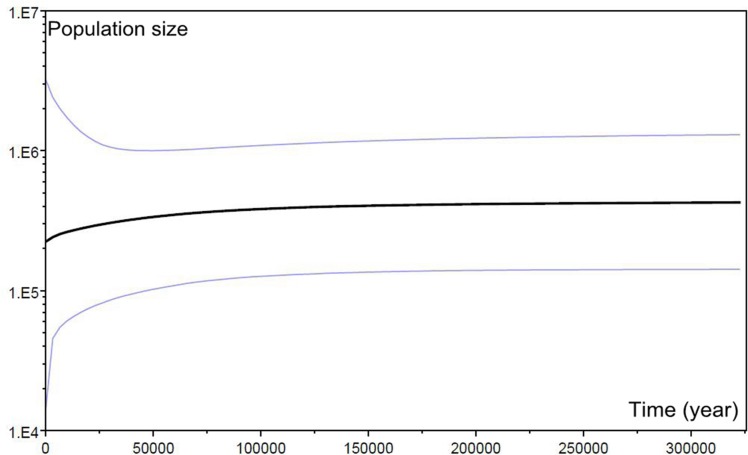
Bayesian skyline plot based on cpDNA for the effective population size fluctuation throughout time. black line, median estimation; area between gray lines, 95% confidence interval.

The mismatch distributions under the sudden expansion model for all the populations were multimodal (Fig. 4), with an SSD of 0.07017 (P = 0.08833) and a HRag of 0.14270 (P = 0.14333), indicating no recent population expansion. This conclusion was also supported by the results of the Neutrality Test, Tajima’s *D* 3.09754 (P < 0.01), Fu and Li’s D* 1.16049, Fu and Li’s F* 2.09321 (P < 0.01), Fu’ *F*s 5.672, Tajima’s *D* and Fu and Li’s F* were all positive values. According to the variation pattern in cpDNA, the Bayesian skyline plot was reconstructed. It showed that the demographic scenario for population of *C*. *multipinnata* was a long history of constant population size, followed by a decline over the last 50,000 years with no subsequent expansion (Fig. 5).

### Genetic diversity of microsatellite loci

Only the locus Cha5 was in the positive selection and it did not reach the significant level of the Fst-outlier. The other sixteen loci fell under the neutral selection. Some of the loci in populations deviated from the Hardy-Weinberg expectations (Table C in S1 File) due to the deficiency of the heterozygotes. All 17 microsatellite loci used for the further analysis.

All 17 microsatellite loci were polymorphic in *C*. *multipinnata*, and a total of 199 alleles were identified. The total number of alleles (N_T_) of each population varied from 71 (HY) to 88 (GLJ) (Table 3). The two populations in Vietnam (YB, HY) have more private alleles (A_P_) than that in the three populations in China (Table 3). The mean number of alleles (N_A_) and the effective number of alleles (N_E_) for each population ranged from 4.176 to 5.176 and 2.314 to 3.197, respectively (Table 3). The observed heterozygosity (Ho) and expected heterozygosity (H_E_) were 0.367 to 0.410 and 0.449 to 0.551, respectively (Table 3). The percentage of polymorphic loci (PPB) had higher values that varied from 88.24% to 100% (Table 3).

**Table 3:** Genetic variability of microsatellites within *C*. *multipinnata* populations.

Pop.	N_T_	N_P_	A_R_	N_A_	N_E_	H_O_	H_E_	I	F	PPB (%)
SD	80	15	3.703	4.706	2.314	0.367	0.449	0.910	0.274	100
SBZ	82	8	3.724	4.824	2.580	0.375	0.462	0.943	0.243	94.12
GLJ	88	18	4.070	5.176	2.885	0.407	0.500	1.031	0.199	94.12
HY	71	26	3.382	4.176	2.572	0.378	0.523	0.966	0.315	94.12
YB	82	30	4.253	4.824	3.197	0.410	0.551	1.122	0.207	88.24
Mean	80.6	19.4	3.826	4.741	2.709	0.387	0.497	0.994	0.248	94.12

Differing from the result of the cpDNA sequences analysis, the AMOVA analysis (Table 2) of the 17 microsatellite loci indicated that 70.43% genetic variation occurs within populations. The mean F_ST_ across all loci and populations was 0.29569 (P < 0.0001), and the F_ST_ values for each population (all *P* < 0.0001) ranged from 0.29386 to 0.29761. The Mantel test (Fig. 2B) based on the SSR data also showed that there was a significant positive correlation (P < 0.05) between the genetic distance (GD) and the geographic distance (GGD), suggesting that *C*. *multipinnata* was isolated by distance.

Gene flow between each pair of the five populations is shown in Table 4, and that between the populations GLJ and SBZ was the highest. The gene flow between populations from China and Vietnam was smaller than 1.

**Table 4:** Estimates of gene flow between each pair of the five populations of *C*. *multipinnata*.

	SD	SBZ	GLJ	HY
SBZ	4.9779			
GLJ	2.4589	7.8722		
HY	0.3628	0.3916	0.4160	
YB	0.4389	0.4116	0.4379	1.4188

**Fig 6:**
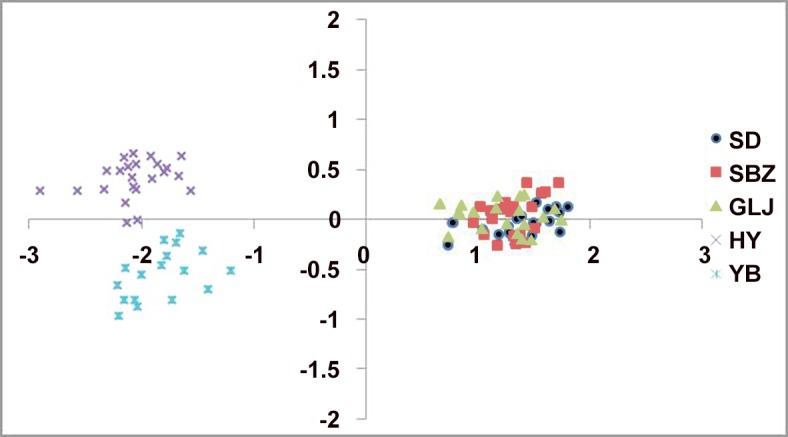
Principal coordinates analysis (PCO) of microsatellites from five populations and 100 individuals of *C*. *multipinnata*.

**Fig 7:**
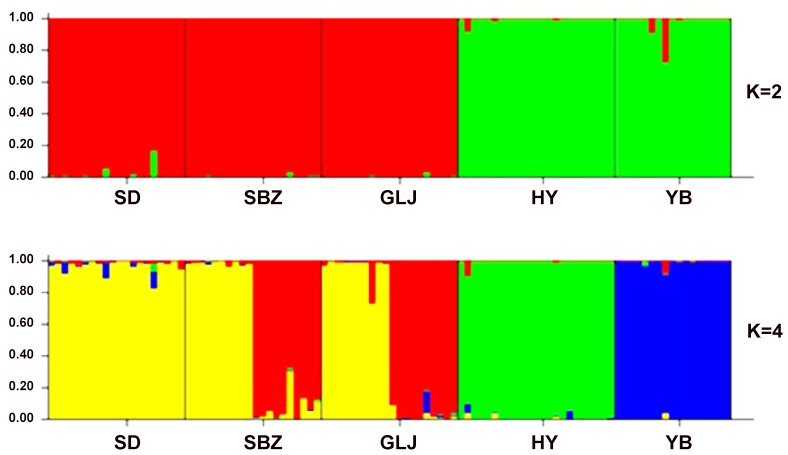
Structure analysis results using the admixture and correlated allele frequencies among populations model.

The two-dimensional PCO (Fig. 6) indicated that these individuals could be approximately divided into two groups. One consisted of the three populations (SD, SBZ and GLJ) in China, and the other consisted of the two populations (HY and YB) in the northern Vietnam. The best and second fit numbers of grouping is inferred as two and four based on the Δ*K* evaluation (Δ*K* = 506.453 when *K* = 2 and Δ*K* = 3.357 when *K* = 4) in the Bayesian clustering analysis, when using the 17 microsatellite loci (Fig. 7). At *K* = 2, as in the PCO analysis, the *C*. *multipinnata* was divided into two major groups. The BARRIER indicated that there was only one barrier, with a 57.65% mean bootstrap value, between the populations in China and Vietnam (Fig. A in S2 File), which means that two clusters came to being among all the individuals of the five populations.

### Population bottleneck analysis of the microsatellite loci

The probabilities of the Wilcoxon test and the sign test in the TPM model showed no significant difference (P > 0.05) except for the YB population (P < 0.01) (Table 5). The allele-distribution mode-shift of all the populations is a normal L-shaped distribution (Table 5). Taken together, bottleneck analysis did not yield evidence of any significant reduction of population size.

**Table 5:** Bottleneck analysis for the five populations of *C*. *multipinnata* based on SSR data.

POP.	T.P.M.		Mode-Shift	G-W index
	Sign Test	Wilcoxon Test		
SD	0.09676	0.10889	L-shaped	0.40903
SBZ	0.40522	0.85364	L-shaped	0.34567
GLJ	0.38307	0.43068	L-shaped	0.33602
HY	0.41521	0.14543	L-shaped	0.31695
YB	0.00866**	0.00025**	L-shaped	0.33259

**: P<0.01, most significant difference; G-W index: Garza-Williamson index, the ratio of number of alleles to range in allele size

However, the Garza-Williamson indices (Table 5) were low, ranging from 0.31695 to 0.40903 across populations, which were lower than the critical value of 0.68 proposed by Garza and Williamson, suggesting past population size reductions.

The effective population sizes (Ne) calculated using the linkage disequilibrium (LD) method with LDNe at the three lowest allele frequency levels with a 95% confidence interval are shown in Table 6. All of the effective population sizes (Ne) in wild (SBZ, GLJ, HY, YB) are smaller than 100, and some are even smaller than 50.

**Table 6:** The effective population size of each population of *C*. *multipinnata*.

population	Ne (Fl = 0.05)	Ne (Fl = 0.02)	Ne (Fl = 0.01)
SD	39.9	151.7	151.7
SBZ	12.9	48.6	48.6
GLJ	7.4	16.7	16.7
HY	67.9	46.6	46.6
YB	21.7	80.5	80.5

Ne: Effective population; Fl: Lowest Alleles Frequency used

## Discussion

The survival of a species relies on adequate genetic diversity to enable adaptation to a changing environment. We used two molecular markers, three chloroplast DNA and 17 nuclear microsatellite loci, to detect the exact genetic variation, differentiation, structure and demographic scenarios of *C*. *multipinnata*. The AMOVA analysis revealed that the variation partition of the three cpDNA intergenic spacers is 92.3% variation among populations and 7.7% within populations, whereas the 17 microsatellite loci indicated that 70.43% genetic variation occurs within populations and 29.57% among populations (Table 2). The cause of this discordance is that the microsatellite mutation rate (3.0×10^-5^ ~ 7.1×10^-4^ substitutions per site per year for synonymous sites [71,72]) is several orders of magnitude greater than that of cpDNA sequence (approximately 1.01×10^-9^ substitutions per site per year for synonymous sites [49,50]), and the microsatellite mutation could be transmitted to the next generation via pollen and seed, so during the same time in a population, the microsatellite DNA could accumulate more variations than cpDNA sequence. For microsatellite DNA evolving neutrally, the amount of polymorphism is expected to be directly proportional to the underlying mutation rate [73]. The two molecular markers have different mutation rate and dispersed mechanism, hence, they could reflect respective genetic events, and it is necessary to choose these two markers to study on the *C*. *multipinnata*.

### Genetic variation and genetic differentiation

Generally speaking, the genetic diversity of *C*. *multipinnata* is moderate in cpDNA sequence. It is higher than that of *C*. *debaoensis* (*atp*B*-rbc*L and *psb*A*-trn*H, H_d_ = 0.49160, H_S_ = 0.179, H_T_ = 0.564, π = 0.00132) [26] but much lower than *C*. *revoluta* (*atp*B*-rbc*L, π = 0.0581) and *C*. *taitungensis* (*atp*B*-rbc*L, π = 0.0181) [74]. For *C*. *simplicipinna*, a unique cpDNA haplotype was detected in all seven populations from both China and Laos [75], so the haplotype diversity (H_d_ = 0.864) and the total diversity (H_T_ = 1.000) is higher than *C*. *multipinnata*, whereas the within-population diversity (H_S_ = 0.076) is lower. The genetic variation within populations for *C*. *multipinnata*, *C*. *debaoensis*, and *C*. *simplicipinna* is low.

In the cpDNA analysis of *C*. *multipinnata*, H2 is the predominant chloroplast haplotype among the eight haplotypes detected in the 60 individuals from six populations. The other five haplotypes in the four populations (SD, SBZ, GLJ and SZD) in China with short branches to H2 represent recently evolved haplotypes. A unique cpDNA haplotype is detected in each of the two Vietnamese populations. The cpDNA genetic diversity in China tends to be higher than that in Vietnam because there are more populations of *C*. *multipinnata* in Yunnan, China, and greater gene flow among them.

*Cycas multipinnata* was characterized by isolation by distance (IBD), i.e., a significant positive correlation (P < 0.05) was detected between genetic distance (GD) and geographic distance (GGD), which was addressed by the accordant results of the Mantel test based on the cpDNA and SSR data.

As with most Cycads, the dioecious *C*. *multipinnata* plants are pollinated by insects, primarily by beetles [76]. Seeds disperse by their own gravity beside the maternal individuals or by rodents that are attracted by the fleshy sarcotesta of cycads [77]. The current gene flow between extant populations is restricted due to the geographical isolation and the low seed dispersal. Thus, the levels of gene flow (Nm, Table 4) between populations cannot be interpreted as current gene flow between populations; they represent either ancient migratory events or shared ancestral polymorphism. So *C*. *multipinnata* has a high genetic differentiation (F_ST_ = 0.92304 based on the cpDNA data; F_ST_ = 0.29569 based on 17 microsatellite loci) according to the Wright’s criterion [78], which an F_ST_ value greater than 0.25 would indicate that there is significant genetic differentiation among populations. Moreover, high genetic differentiation has been detected in many cycads, such as *C*. *debaoensis* (F_ST_ = 0.80102, based on the data of *atp*B*-rbc*L and *psb*A*-trn*H) [26] and *C*. *simplicipinna* (F_ST_ = 0.987, based on the data of *psb*A*-trn*H and *trn*L-*trn*F, F_ST_ = 0.26 based on 16 microsatellite loci) [75]. In contrast with the three inland *Cycas* species above, *C*. *revoluta* and *C*. *taitungensis*, which are coastal or island distributed, possess high genetic variation and low genetic differentiation between populations [74]. One possible reason for this is that the glaciation had different effects on the *Cycas* species occurring inland and on islands. In detail, during the glacial period, migration corridors across islands may have formed that enhanced the gene flow, whereas on land, the environmental niche of *Cycas* plants became restricted to isolated refuges, resulting in limited gene flow. Another reason may be that different *Cycas* species have different lengths of evolution history; for example, the most recent common ancestor (TMRCA) of *C*. *revolute* and *C*. *taitungensis*is is estimated at 327.3 MYA in mtDNA and 204.0 MYA in cpDNA [74]. In a longer evolutionary history with more glaciations, *C*. *revoluta* and *C*. *taitungensis* experienced more complex in-range expansion and contraction fluctuations in population size.

### Late Pleistocene divergence and population contraction

The genealogical haplotype network and topology of the Bayesian tree based on the data of the combined cpDNA sequences showed that *C*. *multipinnata* can be divided into two groups: one included the populations (SD, GLJ, SBZ and SZD) distributed in China and the other included the two populations (HY and YB) that occurred in northern Vietnam. The same result was obtained from three different analyses of the 17 microsatellite loci. First, in the principal coordinate analyses (PCO), 100 individuals from the five populations (SD, GLJ, SBZ, HY and YB) were spilt into two parts: one included the three populations (SD, GLJ and SBZ) in China, and the other contained the remaining two populations in Vietnam. Second, in the Bayesian clustering analysis using the STRUCTURE version 2.3 software, it was strongly recommended that the five populations should be divided into the same two groups suggested in the PCO analysis. Finally, the BARRIER analysis also showed that only one barrier existed between the populations in China and Vietnam. Overall, these analysis based on two different markers reached the same conclusion: *C*. *multipinnata* has a distinct structure, and it contains the China clade and Vietnam clade. The date of the most recent common ancestor (TMRCA) between the clades, based on the cpDNA data is approximately 1.0307 million years ago (MYA) i.e., during the Pleistocene.

The Bayesian skyline plot of cpDNA showed that the *C*. *multipinnata* population size experienced a significant reduction approximately 50,000 years ago (Fig. 5), and this result was supported by the microsatellite-based Garza-Williamson index (Table 5). It is likely that the conditions during the Quaternary glacial periods substantially affected the distribution and genetic structure of *C*. *multipinnata* populations as the temperature fluctuated [79]. Comparing the annual temperature at the fossil locality in northern China (Liaoning, 42° North) with that of the present distributions of cycads, we find that the current distribution of cycads is strongly affected by climate changes. Conversely, the evidence from the molecular analyses supported that both the time of the two clade divergence and the population contraction of *C*. *multipinnata* are associated with the severe climatic oscillation during the Quaternary glaciations. Similarly, that Quaternary glaciations played an important role in the population demographic histories has also been indicated in other *Cycas* species. For example, it was estimated that the TMRCA of different chloroplast haplotypes in *C*. *debaoensis* is approximately 2.66 MYA [26], for *C*. *simplicipinna*, it is 2.682 MYA in cpDNA, and 1.429 MYA according to the ITS4-ITS5 of nrDNA data [75]. The estimated divergence time of the living *C*. *multipinnata*, *C*. *debaoensis* and *C*. *simplicipinna* is could thus date to the Pleistocene. Additionally, a population contraction event of all three species has been detected in the last 50,000 years. Thus, the extant *C*. *multipinnata* populations in China and Vietnam represent the glacial refuges owning the high levels of nucleotide diversity in the cpDNA and the highest number of private haplotypes. Anthropogenic disturbances in the last three decades, such as large scale deforestation, road construction and over exploiting, merely accelerated the decrease in population size.

### Conservation suggestions

There are only six small and fragmented populations of *C*. *multipinnata* remaining; the population SZD has only five individuals. During the fieldwork, we found there are few seedlings and coning plants (particularly for female individuals) due to overexploitation and habitat destruction. Population size is the most important factor among the five criteria to identify threatened species in IUCN [80]. It is believed that effective population size (Ne) of 50 individuals is the minimum to maintain sufficient allelic richness, and the effective population size of 500 individuals is barely sufficient to maintain the genetic variation of quantitative characteristics within populations and the adaptive ability for future environmental change [81]. When 0.02 is used as the lowest allele’s frequency used, the Ne of the YB population is only 80.5, which is greater than the minimum effective population size of 50. The Ne of population SBZ, GLJ and HY is 48.6, 16.7 and 46.6, respectively (Table 6). What is worse, both the Bayesian skyline plot of cpDNA and the microsatellite-based Garza-Williamson indices suggest a reduction in population size. Thus, it is rather urgent to take measures to protect this species.

*In situ* conservation is one important measure to take because the entire gene pools are preserved in their native habitat. Considering the limitation of the wild *C*. *multipinnata* resource and the apportionment of genetic diversity, we should regard all six populations as Management Units (MUs). In addition to public education and legal constraints to curtail overexploitation and habitat destruction, a long-term recovery of *C*. *multipinnata* package should be launched with the active participation of local people.

*Ex situ* conservation is an insurance policy that can be carried out with living plants cultivated in nature reserves or botanic gardens. Because *Cycas* could propagate via vegetative techniques [77], the germplasm collection objects could be basal offsets (suckers) or seeds of *C*. *multipinnata* from as many populations as possible to increase the genetic diversity.

Considering the main impact due to the population contraction is the low genetic variation and low gene flow between populations for both the *ex situ* and *in situ* conservation of *C*. *multipinnata*, we should increase the genetic diversity of the plant material during forest management. Of course, scientific research on *C*. *multipinnata*’s environment and pollination biology should also be carried out in time.

## Conclusions

The historical divergence between the main genetic clusters of *C*. *multipinnata* took place approximately 1.0307 MYA, namely in the Pleistocene. The reconstruction of the population demographic history of *C*. *multipinnata* indicates that over the last 50,000 years, this species underwent a population contraction, with no subsequent expansion. The severe climate oscillation during the Pleistocene is a factor that contributed to the currently isolated geographical distribution of *C*. *multipinnata*. Our study revealed that the extant *C*. *multipinnata* displays low genetic diversity within populations and high genetic differentiation among populations in cpDNA sequence. Overall, the pattern of genetic variation within and among populations in *C*. *multipinnata* was related to the geographic distance. Practical measures should be launched immediately to protect the Endangered *C*. *multipinnata*.

## Supporting Information

S1 File Contains supporting Tables A, B, C. **Table A.** Information of 17 microsatellite loci used to study the population genetics. **Table B.** Variable sites from the three cpDNA combined sequence in *Cycas multipinnata*. **Table C.** P-value of Hardy-Weinberg equilibrium test for the five populations of *C*. *multipinnata*.
(DOCX)

S2 File Contains Figure A. The boundaries detected using the BARRIER program based on matrices of Nei’s (1983) unbiased genetic distance.
(DOCX)
