# Supplementary material for: The Historical Demography and Genetic Variation of the Endangered Cycas multipinnata (Cycadaceae) in the Red River Region, Examined by Chloroplast DNA Sequences and Microsatellite Markers
Source: PLoS One. 2015 Feb 17;10(2):e0117719. doi: 10.1371/journal.pone.0117719 (PMC4331093; doi:10.1371/journal.pone.0117719)
Supplement: S1 File — Table A. Information of 17 microsatellite loci used to study the population genetics. Table B. Variable sites from the three cpDNA combined sequence in Cycas multipinnata. Table C. P-value of Hardy-Weinberg equilibrium test for the five populations of C. multipinnata. (DOCX) [file pone.0117719.s001.docx]

**Supporting Tables**

**Table A. Information of 17 microsatellite loci used to study the population genetics**

| No. | Loci | Repeat motif | *A*_R_ | *I* | *H*_O_ | *H*_E_ | *F* | References |
| --- | --- | --- | --- | --- | --- | --- | --- | --- |
| 1 | Cpz25 | TG | 8.565 | 1.381 | 0.637 | 0.686 | 0.036 | Zhang et al, 2010 |
| 2 | Cpz26 | AT | 4.569 | 0.919 | 0.251 | 0.519 | 0.513 | Zhang et al, 2010 |
| 3 | Cha02 | CT | 5.707 | 0.866 | 0.267 | 0.412 | 0.435 | Zhang et al, 2009 |
| 4 | Cha05 | CT | 2.162 | 0.394 | 0.130 | 0.262 | 0.615 | Zhang et al, 2009 |
| 5 | Cha06 | CT | 3.109 | 0.575 | 0.298 | 0.330 | 0.147 | Zhang et al, 2009 |
| 6 | Cha07 | CT | 11.101 | 2.028 | 0.820 | 0.827 | 0.011 | Zhang et al, 2009 |
| 7 | Cha08 | AG | 5.885 | 0.774 | 0.326 | 0.389 | 0.203 | Zhang et al, 2009 |
| 8 | HL03 | AG | 7.65 | 1.201 | 0.388 | 0.568 | 0.273 | Li et al, 2009 |
| 9 | HL08 | TTC | 4.663 | 0.703 | 0.320 | 0.355 | 0.227 | Li et al, 2009 |
| 10 | CY270 | TC | 2.662 | 0.450 | 0.207 | 0.279 | 0.407 | Angelica et al,2008 |
| 11 | CY280 | AT | 9.796 | 1.747 | 0.459 | 0.776 | 0.401 | Angelica et al,2008 |
| 12 | G45 | TC | 3.93 | 0.745 | 0.445 | 0.431 | -0.032 | Yang et al, 2008 |
| 13 | G46 | TC | 2.371 | 0.306 | 0.130 | 0.163 | 0.263 | Yang et al, 2008 |
| 14 | E001 | CA | 5.583 | 1.238 | 0.647 | 0.662 | 0.024 | Yang et al, 2008 |
| 15 | E004 | AT | 7.731 | 1.470 | 0.490 | 0.703 | 0.313 | Yang et al, 2008 |
| 16 | Cha-est01 | AT | 7.891 | 1.437 | 0.512 | 0.678 | 0.212 | Wang et al, 2008 |
| 17 | Cy-Tai11 | CAG | 3.127 | 0.669 | 0.262 | 0.407 | 0.352 | Ju et al, 2011 |

**Table B. Variable sites from the three cpDNA combined sequence in *Cycas multipinnata***

| haplotype *atpB-rbcL* *psbA-trnH* *psbB-psbH* | | | | | | | | | | | | | | |
| --- | --- | --- | --- | --- | --- | --- | --- | --- | --- | --- | --- | --- | --- | --- |
| 360 | 368 | 369 | 488 | 859 | 1063 | 1064 | 1065-1168 | 1100 | 1129 | 1161 | 1188-1196 | 1784 | 1817 |  |
| H 1 | - | T | T | G | T | G | - | І | - | C | A | ІІ | C | C |
| H 2 | - | T | - | T | G | G | - | І | - | C | A | ІІ | C | A |
| H 3 | - | T | - | T | G | G | - | І | A | C | A | ІІ | C | C |
| H 4 | - | T | - | T | G | G | - | І | - | C | A | ІІІ | C | A |
| H 5 | - | T | - | T | G | G | - | І | - | C | A | ІІ | C | C |
| H 6 | - | T | - | T | G | G | - | І | - | C | A | - | C | A |
| H 7 | T | - | - | G | T | - | - | - | - | T | T | ІІ | A | C |
| H 8 | T | - | - | G | T | A | T | І | - | T | T | ІІ | A | C |

І: CCAT; ІІ: TTTTTATT; ІІІ: AATAAAAA

**Table C. P-value of Hardy-Weinberg equilibrium test for the five populations of *C. multipinnata***

| loci | SD | SBZ | GLJ | HY | YB |
| --- | --- | --- | --- | --- | --- |
| Cpz25 | 0.3944 | 0.0782 | 0.0070** | 0.1951 | 0.0002*** |
| Cpz26 | 0.0069** | 0.0000*** | 0.0000*** | 0.0296* | 0.4934 |
| Cha02 | 0.0001*** | 0.0024** | 0.0000*** | - | - |
| Cha05 | 0.2797 | 0.0081** | 0.0318* | 0.0222* | - |
| Cha06 | 0.0061** | 1.0000 | 0.0335* | 0.5846 | 1.0000 |
| Cha07 | 0.0828 | 0.0045** | 0.0457* | 0.1917 | 0.1577 |
| Cha08 | 0.3533 | 1.0000 | 0.0113* | 0.1955 | 0.3996 |
| HL03 | 1.0000 | 0.0000*** | 0.0000*** | 0.0000*** | 0.1451 |
| HL08 | 0.0243* | 0.0256* | 1.0000 | 0.0689 | 0.5569 |
| CY270 | 0.1520 | 0.0020** | - | 1.0000 | 0.0668 |
| CY280 | 0.0822 | 0.0076** | 0.0113* | 0.0000*** | 0.0000*** |
| G45 | 0.0020** | 0.2785 | 0.0886 | 1.0000 | 0.1879 |
| G46 | 0.0297* | - | 0.0770 | 0.0235* | 1.0000 |
| E001 | 0.4005 | 0.4122 | 0.0282* | 0.2541 | 0.0125* |
| E004 | 0.4653 | 0.2494 | 0.0003*** | 0.0003*** | 0.0000*** |
| Cha-est01 | 0.0015** | 0.0451* | 0.0227 | 0.0000*** | 0.0001*** |
| Cy-Tai11 | 0.0858 | 0.0003*** | 0.0001*** | 0.4115 | 1.0000 |

-: monomorphic; *: P < 0.05; **: P < 0.01; ***: *P* < 0.001
