# Supplementary material for: The Historical Demography and Genetic Variation of the Endangered Cycas multipinnata (Cycadaceae) in the Red River Region, Examined by Chloroplast DNA Sequences and Microsatellite Markers
Source: PLoS One. 2015 Feb 17;10(2):e0117719. doi: 10.1371/journal.pone.0117719 (PMC4331093; doi:10.1371/journal.pone.0117719)
Supplement: S2 File — The boundaries detected using the BARRIER program based on matrices of Nei’s (1983) unbiased genetic distance. (DOCX) [file pone.0117719.s002.docx]

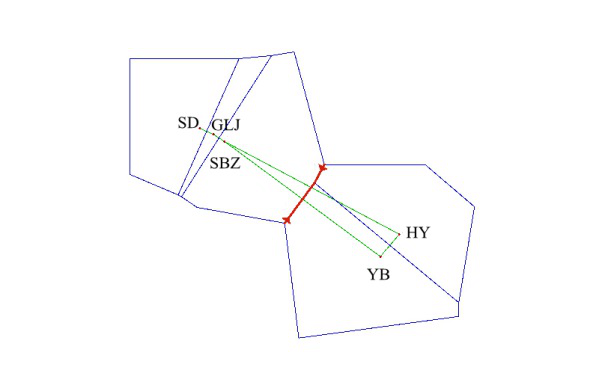


Fig. A **The boundaries detected using the BARRIER program based on matrices of Nei’s (1983) unbiased genetic distance**
